# Leaf physiological and morphological constraints of water-use efficiency in C_3_ plants

**DOI:** 10.1093/aobpla/plad047

**Published:** 2023-07-31

**Authors:** Peter Petrík, Anja Petek-Petrik, Mohammad Mukarram, Bernhard Schuldt, Laurent J Lamarque

**Affiliations:** Karlsruhe Institute of Technology (KIT), Institute of Meteorology and Climate Research-Atmospheric Environmental Research (IMK-IFU), Kreuzeckbahnstraße 19, 82467 Garmisch-Partenkirchen, Germany; Institute of Botany, Czech Academy of Sciences, Lidická 971, 602 00 Brno, Czech Republic; Department of Phytology, Faculty of Forestry, Technical University in Zvolen, T.G. Masaryka 24, 960 01 Zvolen, Slovakia; Chair of Forest Botany, Institute of Forest Botany and Forest Zoology, Technical University of Dresden (TUD), Pienner Str. 7, 01737 Tharandt, Germany; Département des Sciences de l’environnement, Université du Québec à Trois-Rivières, Trois-Rivières, QC G8Z 4M3, Canada

**Keywords:** Crown architecture, leaf anatomy, mesophyll conductance, minimal conductance, respiration, rubisco, stomata, WUE

## Abstract

The increasing evaporative demand due to climate change will significantly affect the balance of carbon assimilation and water losses of plants worldwide. The development of crop varieties with improved water-use efficiency (WUE) will be critical for adapting agricultural strategies under predicted future climates. This review aims to summarize the most important leaf morpho-physiological constraints of WUE in C_3_ plants and identify gaps in knowledge. From the carbon gain side of the WUE, the discussed parameters are mesophyll conductance, carboxylation efficiency and respiratory losses. The traits and parameters affecting the waterside of WUE balance discussed in this review are stomatal size and density, stomatal control and residual water losses (cuticular and bark conductance), nocturnal conductance and leaf hydraulic conductance. In addition, we discussed the impact of leaf anatomy and crown architecture on both the carbon gain and water loss components of WUE. There are multiple possible targets for future development in understanding sources of WUE variability in plants. We identified residual water losses and respiratory carbon losses as the greatest knowledge gaps of whole-plant WUE assessments. Moreover, the impact of trichomes, leaf hydraulic conductance and canopy structure on plants’ WUE is still not well understood. The development of a multi-trait approach is urgently needed for a better understanding of WUE dynamics and optimization.

## Teaser

Climate-change-induced evaporative demand will impact carbon assimilation and water losses in plants. Developing water-efficient plant varieties is crucial. This review summarizes leaf morpho-physiological constraints on water-use efficiency (WUE) in C3 plants, highlighting knowledge gaps. The understanding of WUE dynamics needs further exploration of residual water losses, respiratory carbon losses, trichomes, leaf hydraulic conductance, and canopy structure. A multi-trait approach is needed for understanding WUE optimizing in plants.

## Introduction

Water-use efficiency (WUE) reflects a balance between carbon gain and water loss in plants, introduced more than 100 years ago by Briggs and Shantz (1913). Since then, multiple ways and methods to assess WUE at a different level of organization and temporal resolution were developed and conceptualized (Vadez *et al.* 2014, 2023; Hatfield and Dold 2019; Brendel 2021). Two WUE parameters reflect a momentary state of leaf carbon and water fluxes: intrinsic water-use efficiency (WUE_i_) as a ratio of CO_2_ assimilation rate (*A*_*n*_) to water vapour stomatal conductance (*g*_s_), obtained during gas-exchange measurements at leaf level (Petrik *et al.* 2022*a*). Another closely related variant, instantaneous WUE_i_, is calculated as a ratio of *A*_*n*_ and leaf transpiration (Bacon *et al.* 2004). Other WUE parameters capture the long-term balance between carbon fixation and transpiratory water losses. Biomass-based indices include whole-plant WUE_bio_ as the ratio of biomass accumulation to cumulative transpiration of the plants (Condon *et al.* 2004; Brendel 2021). Furthermore, yield WUE is usually calculated as crop yield per hectare divided by total transpiration or evapotranspiration (Hatfield and Dold 2019; Zahoor *et al.* 2019). The use of growth-based WUE calculated as the ratio of annual basal area increment and cumulative annual transpiration is used in dendrobiology (Szatniewska *et al.* 2022). Moreover, the carbon isotope ratio (δ ^13^C) has been extensively used as a proxy of long-term WUE_13C_, because of the preference for the lighter isotope during physical and chemical processes involved in CO_2_ uptake and assimilation (Farquhar *et al.* 1989; Frank *et al.* 2015; Ma *et al.* 2023). Ecosystem-wide WUE derived from eddy-covariance measurements (WUE_GPP_) is a ratio between gross primary production (GPP) of the ecosystem and total cumulative transpiration or evapotranspiration (Yi *et al.* 2019). WUE_GPP_ can be also derived from remote sensing data as the GPP to evapotranspiration ratio (Ahmadi *et al.* 2019). Overall, the individual-level, long-term (vegetation season) based WUE_bio_ is the most precise assessment of real resource utilization of plants as they capture both assimilatory and respiratory balance with productive and unproductive water losses (Brendel and Epron 2022). WUE_bio_ should thus be more commonly used as the standard WUE estimates in agricultural and plant sciences, instead of the WUE_i_, which is much easier to measure but represents only one point in time.

**Figure 1.**
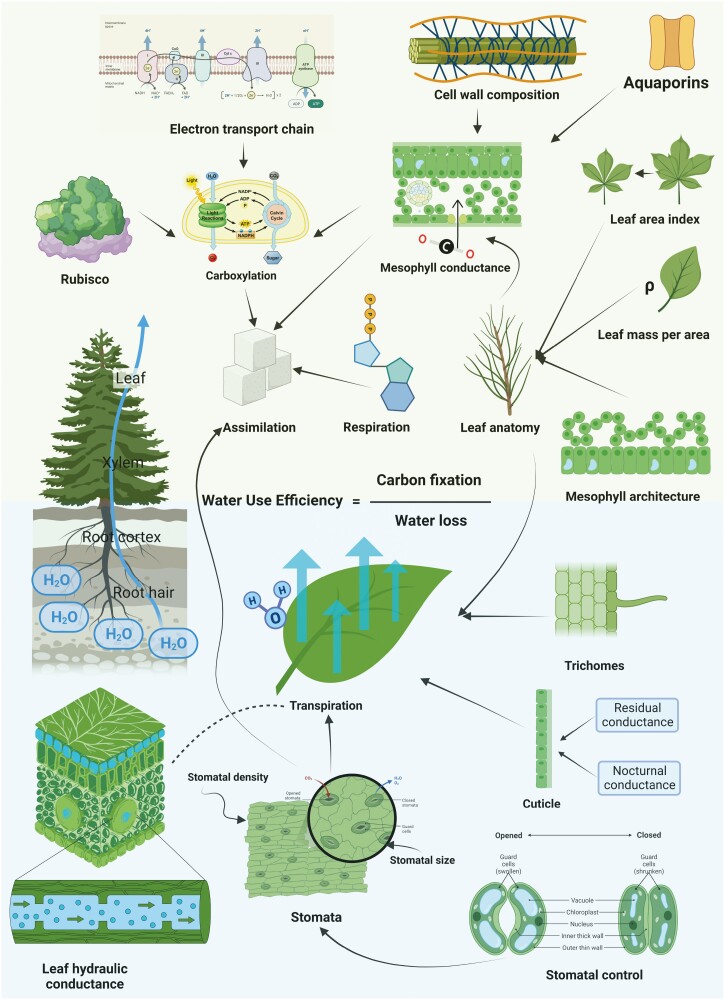
Overview of mechanisms and traits which affect the carbon fixation (upper half) and water loss (lower half) components of water-use efficiency in C_3_ plants. Created with BioRender.com and adapted with Canva.com.

The importance of WUE acclimation in plants is due to raising evaporation demands caused by climate change and possible frequent water-deficit stress during seasonal droughts (Ponce-Campos *et al.* 2013; Schuldt *et al.* 2020). Plants with higher WUE will have a competitive advantage in natural ecosystems and economic significance for agricultural production. The momentary WUE_i_ of plants can be improved either by lower transpiration losses or higher efficiency of carbon assimilation (Flexas *et al.* 2016; Hatfield *et al.* 2019). Understanding of constraining factors of WUE is crucial for crop optimization efforts and the correct assessment of adaptive responses of plant communities (Quan *et al.* 2020; Kang *et al.* 2021). WUE variability is affected by multiple morphological and physiological traits (Figure 1). The size and density of stomata affect the maximal stomatal conductance and stomatal responsiveness to environmental changes (Nunes *et al.* 2022; Pitaloka *et al.* 2022). As stomatal morphology and anatomy can be altered with biotechnological methods for improved WUE, it is a great target for future research (Caine *et al.* 2019; Li *et al.* 2020). The responsiveness of stomata to fluctuating light and drought can also improve long-term WUE_bio_ (Xylogiannis *et al.* 2020; Zhao *et al.* 2021*a*). Several studies have found a negative correlation between WUE estimates and leaf hydraulic conductance (Wedegaertner *et al.* 2022; Barrera-Ayala *et al.* 2023; Liu *et al.* 2023), but these findings are still inconclusive (Corcuera *et al.* 2012; Sellin *et al.* 2014; Jin *et al.* 2016) and we need a better causal explanation of the relationship. Another important constraint of WUE is the mesophyll conductance (*g*_m_) of CO_2_ towards Rubisco (Flexas *et al.* 2016; Zhu *et al.* 2021). Maximization of the *g*_m_/*g*_s_ ratio was suggested as a possible goal for improving WUE of crops (Flexas *et al.* 2013*a*; Fullana-Pericàs *et al.* 2017). The next step of WUE improvement is an optimization of Rubisco carboxylation efficiency (Flexas *et al.* 2016). The long-term WUE_bio_ enhancement could be further achieved by the reduction of respiratory losses and residual water losses during night or drought (Escalona *et al.* 2012; Coupel-Ledru *et al.* 2016). Finally, leaf anatomy, which influences both mesophyll conductance CO_2_ and transpiratory losses, can also alter plant WUE (Bramley *et al.* 2013; Trueba *et al.* 2022).

The objective of this review paper was to summarize various morphological and physiological factors, which influence WUE in plants, as a stepping stone for a more holistic approach to the multi-factor assessment of WUE constraints (Figure 1). We also focused on identifying under-represented physiological and morphological traits in current research, which are needed for understanding WUE optimization in plants. Moreover, this review focuses specifically on WUE_i_, WUE_13C_ and WUE_bio_ to provide the most possibly concise overview of this complex topic at a similar spatial scale. It is worth pointing out that environmental factors such as water availability (Amitrano *et al.* 2019; Zhao *et al.* 2021*b*), soil structure (Hatfield *et al.* 2001; Rabarijaona *et al.* 2022), air pollution (Hatfield *et al.* 2001; Rabarijaona *et al.* 2022) and nutrients (Dijkstra *et al.* 2016; Gharun *et al.* 2021; Song *et al.* 2022) can also have a significant impact on WUE. However, this falls beyond the scope of the study and is therefore not further discussed.

## Water Side of WUE

### Stomatal density and trichomes

Plants can influence their transpiratory losses and therefore potentially their WUE via stomatal regulation (Hetherington and Woodward 2003; Bertolino *et al.* 2019). The stomatal adjustment could include changes in stomatal density (SD), stomatal anatomy (size, shape) and stomatal control mechanisms (Sack and Buckley 2016; Petrik *et al.* 2022*b*). Multiple recent studies, which used genetic manipulation methods to alter SD, have reported improved WUE_i_ connected to the reduction of SD. A genetic manipulation (*EPF2OE*) approach in a study by Franks *et al.* (2015) led to *Arabidopsis* mutants with lower SD that showed higher WUE_i_ and long-term WUE_13C_ due to lower stomatal conductance of water vapour (*g*_s_) but unchanged photosynthetic capacity. Similarly, a combination of high-yield rice cultivars with overexpressed OsEPF1 epidermal patterning factor (EPF) led to a reduction of SD, lower *g*_s_, improved WUE_i_ and overall drought tolerance (Caine *et al.* 2019). The EPF overexpression in bread wheat has led to similar results of reduced SD and improved WUE_i_, without yield losses (Dunn *et al.* 2019). Guo *et al.* (2019*a*) have reported the genetic pathway of EDT1/HDG11, ERECTA, and E2Fa loci, which regulates WUE_i_ of *Arabidopsis* via modulation of SD. Overexpression of *SlTLFP8* (Tubby-like F-box protein 8) reduced SD by 10–20 % in tomatoes and was connected to enhanced WUE_i_ (Li *et al.* 2020). Similarly, repression of *PuGTL1* via Pu-miR172d overexpression led to a reduction of SD and higher WUE_i_ in *Populus ussuriensis* (Liu *et al.* 2021). On the other hand, overexpression of STOMAGEN led to higher SD, greater photosynthetic activity (+30 %), but also greater transpiration (+100 %), which resulted in reduced WUE_i_ (Tanaka *et al.* 2013). Contrary, the study by Bhaskara *et al.* (2022) also reported a positive relationship between SD and WUE_bio_ derived from natural variation in *Arabidopsis* accessions. Moreover, other leaf structures such as trichomes (trichomes/SD ratio) can play a significant role in WUE_i_ and WUE_bio_ enhancement via lower transpiratory losses due to leaf–air boundary layer resistance (Mo *et al.* 2016; Galdon-Armero *et al.* 2018). For example, Chen *et al.* (2022) observed a doubling in trichome density and a decline in *g*_s_ by 85 % between droughted and well-watered *Shepherdia × utahensis* plants. Single gene manipulation efforts, such as *EPF2OE,* could have negative pleiotropic effects on other metabolic processes and should be further explored to avoid these negative side effects (Flexas *et al.* 2016; Husaini *et al.* 2022). It seems that the reduction of SD for improving WUE_i_ and WUE_13C/bio_ could be a viable option for plant breeding initiatives. Additionally, the incorporation of further leaf structures, such as trichomes, in combination with SD can improve our understanding of WUE constraints.

### Stomatal size and responsiveness

Stomatal control mechanisms include reaction to atmospheric vapour pressure deficit (Grossiord *et al.* 2020), plant water potential (Buckley 2005, 2019; Dayer *et al.* 2020), light conditions (Lawson *et al.* 2010; McAusland *et al.* 2013) and CO_2_ concentration (Franks and Beerling 2009). Photosynthetic activity of C_3_ plants can adjust in seconds to changes in irradiance, but the lag in stomatal responses limits the CO_2_ uptake and therefore constrains photosynthesis and limits WUE (Lawson *et al.* 2012). Several studies have reported that smaller stomata respond faster than larger stomata to changes in environmental conditions (Drake *et al.* 2013; Lawson *et al.* 2014; Kardiman and Raebild 2018; Durand *et al.* 2019). Faster stomatal response in the study by Lawson *et al.* (2014) has been linked to higher WUE_i_ values under naturally changing irradiance levels. Theoretical maximal stomatal conductance (*g*_max_) showed a negative correlation with stomatal size, but smaller stomata showed faster response time to variable irradiance in five *Banksia* species (Drake *et al.* 2013). A study by Lei *et al.* (2023) also found that larger stomata of domesticated rice showed slower response time to fluctuating light and overall lower WUE_13C_. The genetic manipulation study in rice has found that mutants with small stomatal size showed higher WUE_i_, in comparison to mutants with greater stomatal size (Pitaloka *et al.* 2022). Des Marais *et al.* (2014) found that *Arabidopsis* genotypes with larger stomata due to AtMPK12 substitution showed lower WUE_i_ compared with the common allele. The improved WUE_i_ of wheat cultivars under water-deficit stress was linked to smaller stomatal size, lower SD and reduced transpiration rates (Li *et al.* 2017). A study by Amitrano *et al.* (2021) showed that a 49 % increase in WUE_i_ and WUE_bio_ of lettuce has been associated with a reduction of stomatal size under different vapour pressure deficit (VPD) treatments. Moreover, drought stress exposure inhibited stomatal development (smaller stomata) and increased the WUE_i_ in cotton (Dubey *et al.* 2023). On the other hand, a study by Xiong and Flexas (2020) on ferns, gymnosperms and angiosperms found a negative correlation between stomatal size and *g*_m_, therefore possibly limiting WUE. A comparison of *Quercus robur* genotypes has found a positive correlation between guard cell length and WUE_13C_, contradicting the majority of results suggesting that smaller stomata promote higher WUE_13C_ (Roussel *et al.* 2009). Liu *et al.* (2018*a*) have found a quadratic relationship between stomatal size and WUEi at the community level, across forest ecosystems along the latitudinal transect, with an optimal stomatal size of approximately 400 μm^2^. Smaller stomatal size could be connected to higher WUE in plants, presumably due to faster response to environmental conditions. Nevertheless, there is probably an optimal stomatal size and further reduction can be detrimental due to CO_2_ limitations of photosynthesis.

### Stomatal control and light sensitivity

Excessive water loss under an impaired state of photosynthetic apparatus (drought, salinity stress) can negatively affect the WUE of plants. Timely stomatal closure is then another major component of WUE optimization of plants under water-deficit stress (Yang *et al.* 2016; Hartmann *et al.* 2021). A study by Yi *et al.* (2019) showed that WUE_13C_ of isohydric species was generally more sensitive to environmental change due to their conservative water potential regulation strategy than WUE_13C_ of the anisohydric species and increased significantly with rising VPD during periods of water stress. The accumulation of abscisic acid (ABA), which drives the stomatal closure of plants under water deficit, can be considered a key factor for both WUE_i_ and WUE_13C/bio_ improvement in plants (Negin and Moshelion 2016; Guo *et al.* 2019*b*; Mukarram *et al.* 2021). Plants capable of fine-tuning their stomatal control with ABA can possess an enhanced WUE_i_ with sustained biomass or yield gains (Yoo *et al.* 2009; Yao *et al.* 2021). Improved WUE_i_ in the presence of elevated ABA levels has been demonstrated in transgenic *Arabidopsis* (Zhang *et al.* 2008) and tomato (Thompson *et al.* 2007; Lamarque *et al.* 2020). Exogenous application of ABA showed enhanced WUE_i_ and WUE_13C_ in *Populus davidiana* (Li *et al.* 2004) and *Marsilea crenata* fern (Tai-Chung *et al.* 2020). French bean and sugar beet plants pretreated with ABA also showed improved WUE_i_ under water-deficit stress (Pospíšilová and Baťková 2004). Enhanced stimulation of ABA signalling of *Arabidopsis* via distinct ABA receptors can result in constitutively high WUE_i_ (Yang *et al.* 2016). WUE_13C_ of *Arabidopsis* and wheat was also enhanced by modulating ABA responses either by using overexpression of specific ABA receptors or deficiency of ABA coreceptors (Yang *et al.* 2019). ABA receptors from *Populus canescens* were stably introduced into *Arabidopsis* in a study by Papacek *et al.* (2019), which led to enhanced WUE_i_. Moreover, overexpression of *PeJAZ2* increased WUE_i_ of poplar under drought stress by regulating ABA signalling rather than ABA synthesis (Rao *et al.* 2023). Partial root-zone drying can generate a root-to-shoot pressure signal from the dry part of the root zone that also promotes stomatal closure via a drop in cell turgor and enhances WUE_i_ via ABA utilization (Davies *et al.* 2002; Pérez-Pérez *et al.* 2012; McAdam and Brodribb 2016; Zhang *et al.* 2018; Xylogiannis *et al.* 2020). These results, therefore, suggest great opportunities for WUE optimization in crops with the use of transgenic methods, breeding efforts and biotechnological tools for ABA utilization.

Stomatal sensitivity to light could be another important determinant of plant WUE_i_ by adjusting the magnitudes of change in *g*_s_ as a function of the environment (Vialet-Chabrand *et al*. 2016). Part of the stomatal response involves the balance between photosynthetic electron transport and carbon reduction either in guard cells, chloroplasts, or in the mesophyll (Messinger *et al.* 2006). Overexpression of *Photosystem II Subunit S* in tobacco led to lower stomatal opening in response to light, which resulted in a 25 % reduction of water loss and improved WUE_i_ (Glowacka *et al.* 2018). The desynchronization of *A*_*n*_ and *g*_s_ can lead to a surplus in transpiration when *A*_*n*_ is low but *g*_s_ is high (e.g. transition from high to low light), hence reducing WUE_i_ (McAusland *et al.* 2016; Coupel-Ledru 2021). The introduction of a blue light-activated K^+^ ion channel, named BLINK1, to *Arabidopsis*, led to a faster reaction of stomatal aperture under both increasing and decreasing irradiance, which ultimately enhanced the plants’ biomass accumulation and WUE_bio_ (Papanatsiou *et al.* 2019). Dynamic plant response to VPD and light fluctuations under natural conditions were suggested to increase plants WUE_bio_ (Gosa *et al.* 2019). Lower stomatal openness and lower *g*_s_ under short-term light transitions led to higher WUE_i_ in chilli pepper treated with “smart glass” compared to the control group (Zhao *et al.* 2021*a*). A study by Li *et al.* (2023) found that overexpression of *OE-PtrVCS2* in *Populus trichocarpa* led to smaller stomatal aperture under drought stress and overall higher WUE_i_ than in the wild type. Greater WUE_i_ of isohydric Pine species has been also linked to lower stomatal openness under increasing light, while anisohydric Oak species behaved more opportunistically with lower WUE_i_ (Renninger *et al.* 2015). Reduction of stomatal openness as a reaction to light changes can probably improve the WUE of plants but can lead to a reduction of the total growth and yield of crops. Nevertheless, improving stomatal response time to changing irradiance levels can improve the plants’ WUE without a negative impact on assimilation and growth.

### Residual and nocturnal conductance

When the stomata are closed (night, drought), plants are still losing water via their cuticle, bark or incompletely closed stomata (Duursma *et al.* 2019; Lintunen *et al.* 2021). Cuticular transpiration has been recognized as a significant factor affecting drought survival rates (Duursma *et al.* 2019) and might affect WUE_13C/bio_ due to residual transpiration (Ni *et al.* 2012; Ávila-Lovera *et al.* 2019). Minimum leaf conductance (*g*_min_) incorporates water loss across the leaf cuticle, bark and through the incompletely closed stomata (Schuster *et al.* 2017; Blackman *et al.* 2019; Duursma *et al.* 2019; Lintunen *et al.* 2021). Minimization of these residual losses during periods of reduced assimilation rate due to stomatal limitations can therefore lead to improved long-term WUE_13C/bio_ (Sevanto 2020). The water loss from leaves of plants under drought is dominated by *g*_min_ after stomatal closure. This has been related to the thickness of the cuticular wax layer, which increases in response to water deficit (Jeffree 2006; Shepherd and Wynne Griffiths 2006; Bueno *et al.* 2020). However, a relationship between the thickness of the cuticular wax layer and *g*_min_ can be insignificant, both within (Anfodillo *et al.* 2002; Bueno *et al.* 2020) and across species (Riederer and Schreiber 2001). The variability of *g*_min_ can be also driven by stomatal morphology (leaky stomata) or chemical composition of cuticle (Duursma *et al.* 2019; Machado *et al.* 2021). In a recent study across 23 genotypes of wheat, cuticular transpiration showed a strong positive correlation with water loss per dry mass unit, which the authors considered as a proxy for WUE_bio_ (Gašparovič *et al.* 2021). A modelling simulation approach by Duursma *et al.* (2019) revealed a theoretical reduction of WUE_i_ under increasing *g*_min_ of plants using the general Ball-Berry model of stomatal conductance. Moreover, hydroponically grown *Festuca arundinacea* exposed to salinity treatment showed enhanced WUE_i_ and lower *g*_min_ compared to the control group (Vandegeer *et al.* 2021). On the other hand, eucalyptus clones under water-deficit treatment showed significant intra-specific differences in cuticular conductance but not in WUE_i_ (Carignato *et al.* 2019). A study by Clarke *et al.* (1991) also found no significant correlation between minimal conductance and long-term WUE_bio_ in wheat under drought stress. The impact of cuticular conductance or *g*_min_ on WUE has not been yet properly quantified and is therefore a great target for future research.

The analogical parameter, nocturnal conductance, is also critical for optimization of long-term WUE_13C/bio_ (Coupel-Ledru *et al.* 2016; Even *et al.* 2019). Excessive water losses during the night (Dawson *et al.* 2007; Forster 2014) decrease long-term WUE as there is no photosynthetic gain during the night. It has been suggested that the low nocturnal conductance of shade-tolerant plant species is consistent with their conservative water-use strategy (Resco de Dios *et al*. 2019). Nocturnal conductance is usually dominated by cuticular transpiration, but incomplete stomatal closure during the night has been observed in C_3_ plants (Caird *et al.* 2007; Escalona *et al.* 2012). Reduction of night transpiration can theoretically improve the WUE_bio_ of crops without growth penalties (Tardieu *et al.* 2022). A study by Dayer *et al.* (2021) has shown that night transpiration was linked more to the specific circadian rhythm of the wine cultivars rather than environmental conditions, suggesting strong genetic control. Night transpiration also had a significant impact on total transpiration and WUE_bio_ in a study by Medrano *et al.* (2017) and was recognized as one of the under-explored factors affecting whole-plant WUE. Nocturnal conductance also showed a significant negative correlation with WUE_bio_ among black poplar genotypes exposed to drought stress (Bogeat-Triboulot *et al.* 2019). Differences in the night transpiration between *Pinus contorta* thinning treatments corresponded to differences in WUE under water-deficit stress (Wang *et al.* 2020). Further quantification of the night transpiration effect on the long-term WUE of plants is needed for a proper understanding of the phenomenon. Selection for plants with low cuticular conductance and conservative stomatal control (avoiding leaky stomata) can greatly improve their WUE and drought resistance.

### Leaf hydraulic conductance

Leaf hydraulic conductance (*K*_leaf_) can be coordinated with higher WUE_i_, as observed in several studies (Fichot *et al.* 2009; Andrade *et al.* 2022; Wedegaertner *et al.* 2022). Nevertheless, it is still unknown if the plants with higher WUE develop smaller xylem vessels causing lower *K*_leaf_ (but greater xylem embolism resistance, cf. Isasa *et al.* 2023) as they have lower hydraulic requirements to maintain leaf gas exchange, or the lower *K*_leaf_ leads to greater WUE by constraining water supply in leaves. *K*_leaf_ is tied to leaf assimilation and stomatal conductance rate in a positive linear fashion (Santiago *et al.* 2004; Sellin *et al.* 2014). Reduction of leaf hydraulic conductance via gene manipulation can lead to lower water losses but is also tied with a proportional reduction of assimilation rates and therefore non-significant changes in WUE_bio_ (Zsögön *et al.* 2015). The environmental response of *K*_leaf_ and its impact on WUE has received more attention in recent studies and has been identified as a major trait that could constrain WUE under changing VPD (Flexas *et al.* 2013a; Xiong *et al.* 2018). However, no consensus has been reached to date regarding the direction of the relationship between *K*_leaf_ and WUE. On one hand, Yao *et al.* (2021) reported that raising WUE_i_ of *Caragana* sp. with decreasing water potential was coordinated with decreasing *K*_leaf_ but also rapid biosynthesis of ABA. The Solanum species with significantly lower *K*_leaf_ showed also significantly higher WUE_13C_ under well-watered conditions (Barrera-Ayala *et al.* 2023), while WUE_i_ of *Ginkgo biloba* was also negatively correlated with *K*_leaf_ (Liu *et al.* 2023). Warming treatment in four subtropical tree species led to higher *K*_leaf_ but lower WUE_13C_ (Wu *et al.* 2020). On the other hand, Jin *et al.* (2016) reported a positive relationship between *K*_leaf_ and WUE_i_ among 10 temperate tree species. Similarly, a positive correlation between WUE_13C_ and *K*_leaf_ was reported for *Pinus pinaster* populations exposed to drought stress (Corcuera *et al.* 2012). Moreover, Sellin *et al.* (2013, 2014) found no significant correlation between WUE_i_ and *K*_leaf_ in birch and aspen trees. In conclusion, the direction of the *K*_leaf_-WUE relationship is unclear, and further work must be conducted to assess whether breeding for lower *K*_leaf_ to reduce water losses possibly leads to improved WUE without a significant reduction of growth. Future experiments with gene manipulation techniques that will not affect other physio-morphological traits are needed to understand the causal link of these correlations.

## Carbon Side of WUE

### Mesophyll conductance

Improving CO_2_ diffusion to the sites of carboxylation without increasing stomatal conductance can enhance WUE_i_. This requires improving mesophyll conductance to CO_2_ (*g*_m_) and it has been proposed that the ratio *g*_m_/*g*_s_ is a relevant breeding trait for improving WUE (Galmés *et al.* 2011; Flexas *et al.* 2013*b*; Tomás *et al.* 2014*a*; Flexas 2016). The *g*_m_ has been recognized as one of the main limiting factors of WUE in both crops (Leakey *et al.* 2019) and tree species (Zhu *et al.* 2021), potentially due to the close coupling of *g*_m_ and *K*_leaf_ as both share the same pathways of water movement in leaves (Flexas *et al.* 2013b; Xiong *et al.* 2017). A close positive relationship has also been observed between *g*_m_ and *g*_s_ although the reason for this remains speculative (Guiliani *et al.* 2013; Barbour and Kaiser 2016). However, a study by Fullana-Pericas *et al.* (2017) showed a strong positive correlation between *g*_m_/*g*_s_ and WUE_i_ in Mediterranean tomato landraces. Similarly, WUEi showed a strong positive correlation with *g*_m_/*g*_s_ in tobacco under chloride nutrient treatments (Franco‐Navarro *et al.* 2019). The variability of *g*_m_ has been linked to leaf anatomy, where cell wall thickness, membrane permeabilities, cytosol and stromal conductance were constraining factors of *g*_m_ (Terashima *et al.* 2011; Tomás *et al.* 2013; Ouyang *et al.* 2017). The cell wall conductance to CO_2_ can be influenced by cell wall thickness, porosity and tortuosity (Evans *et al.* 2009; Ellsworth *et al.* 2018). A study by Roig-Oliver *et al.* (2020) found a strong negative correlation between cellulose and *g*_m_ in grapevine. The hemicellulose to pectin ratio of the cell wall correlated positively with the *g*_m_ of tobacco exposed to drought and salinity stress (Clemente-Moreno 2019). Tholen *et al.* (2008) manipulated the chloroplast arrangement in Arabidopsis and thus modified *g*_m_ through changes in the surface of chloroplasts exposed to the intercellular air spaces (Sc/S). The positive impact of Sc/S on *g*_m_ and *A*_*n*_ has been observed also for Mediterranean oak species (Peguero-Pina *et al.* 2017), rice (Xiong *et al.* 2017) and tobacco (Clarke *et al.* 2021). A recent study by Baillie and Fleming (2020) has found that coordination of stomatal and mesophyll development is crucial for the optimization of *g*_m_ and therefore WUE. Findings to date suggest that certain stomatal development signalling components, such as TMM, ER and STOMAGEN, may be required for interlayer coordination, and that gas exchange may also regulate mesophyll structure (Dow *et al.* 2017). Acclimation of *g*_m_ to changing environmental conditions has been linked to aquaporins and carbonic anhydrase (Flexas *et al.* 2006; Warren 2007). The *g*_m_ can be affected by specific genes (e.g. aquaporin *NtAQP1*, *HvPIP2*, *AtBBX21*) and thus targeted by genetic manipulation of crops (Evans *et al.* 2009). Overexpression of aquaporin genes led to increased *g*_m_ (Hanba *et al.* 2004) and inhibition of lower *g*_m_ in various crops (Flexas *et al.* 2006). Tobacco aquaporin NtAQP1 aids the trans-membrane transport of CO_2_ in plants and thus contributes to the CO_2_ permeability of the plasma membrane of the mesophyll cells (Uehlein *et al.* 2003). Carbonic anhydrase activity has been positively correlated to *g*_m_ (Price *et al.* 1994; Momayyezi and Guy 2017) and chloroplast fraction of *g*_m_ (Gillon *et al.* 2000). Carbon anhydrase accelerates the interconversion of the dissolved inorganic carbon species, CO_2_ and HCO_3_^-^, which helps optimize the initial stages of photosynthesis. A recent study by Gómez-Ocampo *et al.* (2021) found that overexpression of *AtBBX21* led to enhanced *g*_m_ and *J*_max_, coupled with higher WUE in potato plants under drought. Moreover, manipulation of heterotrimeric G protein signalling can improve plants’ WUE_i_ and productivity due to higher *g*_m_ rates under drought conditions (Zait *et al.* 2021). More specifically, the canonical Gα (RGA1) subunit gene of G protein regulated gm in rice, which was reflected in improved photosynthetic capacity and overall WUE (Wang and Botella 2022). The optimization of *g*_m_ and therefore WUE is multifaceted and incorporates multiple organizational levels from cell biochemistry to whole leaf anatomy. There is also great intra-specific variability of *g*_m_ across crops (Tomás *et al.* 2014*a*; Chen *et al.* 2021) and trees (Momayyezi and Guy 2017; Peguero-Pina *et al.* 2017) and therefore, it is a reasonable target for breeding efforts which aim at maximizing WUE. Nevertheless, the practical performance of the population/individual’s selection could be hindered by the low reliability of current *g*_m_ measurements (Pons *et al.* 2009; Lundgren and Fleming 2020). The development of more precise *g*_m_ measurement techniques (Márquez *et al.* 2023) could greatly improve the understanding of WUE constraint by *g*_m_. Furthermore, the strong coupling of *g*_m_ with *K*_leaf_ (Flexas *et al.* 2013; Xiong *et al.* 2017) and *g*_s_ (Guiliani *et al.* 2013; Barbour and Kaiser 2016) might impede efforts to improve WUE_i_ through modification of *g*_m_. As shown by Pathare *et al.* (2023) using rice cell wall mutants, modifying *g*_m_ indeed increases photosynthetic capacity but at the cost of simultaneously increasing *g*_s_, resulting in no overall change in WUE_i_.

### Carboxylation rate

Another target to achieve improved photosynthesis is to improve the biochemical capacity for CO_2_ assimilation, that is, improving the carboxylation efficiency of Rubisco for C_3_ species (Gago *et al.* 2014; Flexas *et al.* 2016). Optimizing the efficiency of RuBP carboxylation by Rubisco has the potential of improving WUE by decreasing the concentration of CO_2_ required to achieve high photosynthetic rates (Carmo-Silva *et al.* 2015). The maximum carboxylase activity of Rubisco (*V*_cmax_) and the capacity for photosynthetic electron transport (*J*_max_) can constrain the WUE from the carbon assimilation side. Maintenance of functional electron transport under drought stress led to higher WUE_i_ in *Magnolia grandiflora* (Vastag *et al.* 2020). Reduction of *V*_cmax_ under ozone treatment caused decoupling of photosynthesis and stomatal conductance, which led to lowered WUE_i_ in rice (Masutomi *et al.* 2019) and poplar clones (Xu *et al.* 2022). *V*_cmax_ and therefore photosynthetic capacity increases with leaf maturation, thus young spring foliage can experience reduced WUE_13C_, which can be critical, especially during spring droughts (Cernusak 2020). Enhanced WUE_i_ of common bean genotypes under heat stress was linked to higher *V*_cmax_ (Suárez *et al.* 2021). Additionally, *V*_cmax_/*g*_s_ ratio has been suggested as a useful trait to characterize WUE_i_ variability (positive correlation) across multiple plant species (Flexas *et al.* 2014). Acclimation of WUE_i_ and WUE_13C_ was coupled to *V*_cmax_ and *J*_max_ across *Arabidopsis* genotypes in a study by Easlon *et al.* (2014). Moreover, the improvement of WUE_i_ in *Brassica juncea* was linked to higher carboxylation efficiency (A/C_i_) under biochar treatment (Silva Gonzaga *et al.* 2019). Photosynthesis and therefore WUE_i_ can be limited by Rubisco and RuBP regeneration, especially under high irradiance conditions (Galmés *et al.* 2014). Plants with simultaneous stimulation of RuBP regeneration and electron transport can improve their WUE_i_ due to better photosynthetic capacity (López-Calcagno *et al.* 2020). Other alternatives to improve the WUE_13C/bio_ would be decreasing photorespiration by means of higher Rubisco efficiency for CO_2_ (Whitney *et al.* 2011; Parry *et al.* 2013) or altering the photorespiratory CO_2_ release by adjusting metabolic pathways in leaves (Peterhansel and Maurino 2011). Total leaf N content shows a significant positive impact on the carboxylation capacity of plants (Wright *et al.* 2003; Paillassa *et al.* 2020). The identification of specific amino acids affecting Rubisco kinetics (Orr *et al.* 2016) may provide suitable targets for improving CO_2_ assimilation and consequently WUE_i_ (Nadal and Flexas 2019). Further exploration of optimization of Rubisco activity can positively influence the WUE of plants without any direct trade-off with growth capacity and yield of crops.

### Respiration

Carbon loss through respiration is another process that decreases WUE_bio_ (Seibt *et al.* 2008; Gago *et al.* 2014; Tortosa *et al.* 2016). Plants with lower maintenance respiration rates can maintain higher WUE_bio_. Moreover, respiration could be considered the main factor behind the gap between WUE_i_ and whole-plant WUE_bio_ (Medrano *et al.* 2017). High respiratory losses were linked to lower WUE_bio_ of C_4_*Miscanthus x gigantus* located in USA drylands (Maleski *et al.* 2019). Greater night-time respiration (i.e. high nocturnal transpiration) has been also recognized as one of the major factors behind the reduction of WUE_bio_ under magnesium deficiency of barley (Tränkner *et al.* 2016). High VPD fluxes led to larger reductions in photosynthesis in comparison to respiration, which decreased the overall productivity and WUE_bio_ of plants from a semi-arid ecosystem (Roby *et al.* 2020). The higher stability of mitochondria and susceptibility of chloroplasts, especially PSII, to abiotic stress can negatively influence the balance between carbon assimilation and respiration towards lower WUE_i_ (Dahal and Vanlerberghe 2017). Root respiration explained around 40 % of WUE_bio_ reduction in both well-irrigated and non-irrigated treatments of grapevine (Tomás *et al.* 2014*b*). Root respiration might be a major component of total plant respiration and thus an important target for further exploration for WUE_bio_ optimization (Escalona *et al.* 2012). Leaf development (maturation) connected with greater respiratory losses could be seen as an additional constraint to long-term WUE_13C_ (Zufferey 2016; Hernández-Montes *et al.* 2019). There is a still lack of precise quantification of day respiration or night-time respiration effect on whole-plant WUE_bio_ and further research is needed. Nevertheless, respiration is connected with plant growth and fruit ripening. Therefore, plant breeding or genetic manipulation efforts that would aim at reducing respiration rates would probably lead to a significant reduction of growth and/or yield. Higher respiratory losses could be also linked to the upregulation of antioxidant systems and artificial reduction of respiration could be therefore defective. The inclusion of respiration for WUE calculation creates a more robust estimate, which improves the correlation with whole-plant WUE_bio_ (Cernusak *et al.* 2007; Zhang *et al.* 2019). For example, Senbayram *et al.* (2015) have shown that the 9.8–48.6 % beneficiary effect of nitrogen fertilization on daytime WUE_i_ was lost when nocturnal stomatal conductance and night-time respiration were taken into consideration. Therefore, the respiratory aspect of carbon balance should not be neglected for correct total plant WUE_bio_ estimates.

## Leaf Anatomy and Plant Crown Architecture

Leaf anatomy can affect the mesophyll diffusion conductance to CO_2_, carboxylation capacity and ultimately WUE in plants (Tomás *et al.* 2013; Carriquí *et al.* 2015). Increasing internal air volume might have positive effects on WUE_i_ (Mediavilla *et al.* 2001), probably due to enhanced internal CO_2_ conductance to the site of carboxylation. Similarly, Guerfel *et al.* (2009) reported more efficient water use associated with thicker palisade parenchyma in olive trees. The leaves’ architecture can influence the WUE_i_ due to variable mesophyll porosity and SD to intercellular airspace volume ratio in coniferous tree species (Trueba *et al.* 2022), and cell wall properties such as cell wall thickness (*T*_cw_) might influence *g*_m_ and thus WUE_i_ (Flexas *et al.* 2021; Pathare *et al.* 2023). Mutant rice populations with higher leaf mass per area (LMA) showed improved whole-plant WUE_bio_ under both control and water-limited conditions (Reddy *et al.* 2020*a*). In the study by Horike *et al.* (2021), WUE_i_ of five shrub species covaried with LMA under drought stress. LMA differences explained WUE_13C_ variance across rice mutants through its influence on carbon gain (Reddy *et al.* 2020*b*). A study by Medrano *et al.* (2009) also reported a positive correlation between WUE_i_ and LMA in Mediterranean herbs and shrubs. Similarly, LMA was positively correlated with WUE_13C_ among trees (*Betula, Larix, Pinus*) in the boreal forest (Ge *et al.* 2022). A thicker leaf can be associated with a thicker boundary layer, which lowers transpiratory losses and ultimately improves WUE_bio_ (Bramley *et al.* 2013). The manipulation of leaf anatomy has been proposed as a potential theoretical target for improving photosynthetic capacity and WUE in plants (Tholen *et al.* 2012). The development of plants with thicker leaves and high internal air volume can theoretically improve their WUE.

Further macro-morphological constraint, which affects the whole-plant WUE_bio_, is plant crown architecture (Christina *et al.* 2016; Medrano *et al.* 2017; McNeil *et al.* 2023). A more complex crown architecture creates shade for inner leaves, which can reduce evaporative demand and therefore improve WUE balance. A positive effect of shading treatment on leaf-level WUE_i_ has been observed for *Actinidia chinensis* (Chartzoulakis *et al.* 1993), *A. deliciosa* (Montanaro *et al.* 2009), *Citrus aurantium* (García-Sánchez *et al.* 2015), *C. sinensis* (Jifon *et al.* 2003; Syvertsen *et al.* 2003), *Coffea arabica* (Liu *et al.* 2018b) and *Fragaria* ×*ananassa* (Cordoba-Novoa *et al.* 2022). It is notable to say that shade leaves are optimized for low irradiance and if exposed to direct sunlight (crown damage) they can show decreased WUE_i_ (Dai *et al.* 2009). Moreover, the leaves of *Pinus taeda* in the lower parts of the crown showed significantly higher WUE_i_ in comparison to the upper part during the peak of the vegetation season (Blazier *et al.* 2004). The WUE_13C_ derived from wood in *Fagus crenata* and *Quercus crispula* showed a positive correlation with tree height, crown depth and crown width (Osada *et al.* 2004). Furthermore, Glenn *et al.* (2015) showed that the less complex pillar form of *Prunus persica* had lower WUE_i_ due to higher canopy transpiration in comparison to the common crown form. Leaf area index (LAI) as an indicator of crown density also shows a positive impact on WUE_bio_ across various terrestrial ecosystem types (Li *et al.* 2018; Luo *et al.* 2022). The raising WUE_bio_ of Alpine grasslands has been also linked to increasing LAI (Ma and Zhang 2022). Nevertheless, higher LAI and therefore greater total transpiration can be detrimental for arid regions where it can have a negative impact on WUE_bio_ (Malone *et al.* 2016). More complex crown architecture and higher LAI can enable plants to optimize and improve their whole-plant WUE due to the shading effect and probably also due to better microclimatic conditions within the crown.

## Conclusion and Future Prospects

The WUE balance of plants is multifaceted and affected at multiple levels of organization from molecular to whole-plant level. The main constraining factors identified in this review were stomatal morphology and control, minimal and nocturnal conductance, mesophyll conductance, carboxylation efficiency, respiration rates, leaf anatomy and crown architecture. The traits are usually analysed in research papers separately or in specific combinations (e.g. stomatal morphology and gas exchange). We suggest that future research should include multi-trait analyses with the aim of WUE optimization, thereby deepening our understanding of the coupling and decoupling of carbon uptake and water-use traits. The technological progress of phenotyping platforms can lead to more robust experimental designs that could handle multi-trait analysis. The night-time transpiration and respiration seem to be under-developed major aspects of long-term WUE optimization, which could be further investigated. The effect of leaf hydraulic conductance and canopy structure on WUE is also not very well understood and can be improved. A better understanding of morpho-physiological constraints of WUE can help us to effectively develop more drought-resilient crop and tree species.

## Sources of Funding

PP was supported by the Federal Ministry of Education and Research, BMBF project BioWaWi grant number 16LW0093.

## Contributions by the Authors

PP conceived the paper idea. PP, APP and MM wrote the first draft. BS and LJL supervised the process and helped with the editing of the manuscript.

## Conflict of Interest Statement

None declared.

## Supporting Information

The following additional information is available in the online version of this article –

## Data Availability

No original data was used in this commentary. The discussion and synthesis are based on already published studies.

## Notes

**Plants, Ecosystems & Climate. Chief Editor:** Mary Heskel
